# Association of COVID-19 Intensity With Burnout and Perceptions of Residency Preparedness Among Medical Students

**DOI:** 10.1001/jamanetworkopen.2023.47957

**Published:** 2023-12-13

**Authors:** Liselotte N. Dyrbye, Danielle Brushaber, Colin P. West

**Affiliations:** 1 Division of General Internal Medicine, Department of Medicine, University of Colorado Anschutz Medical Campus, Aurora; 2 Division of Biomedical Statistics and Informatics, Department of Quantitative Health Sciences, Mayo Clinic, Rochester, Minnesota; 3 Division of General Internal Medicine, Department of Internal Medicine, Mayo Clinic Rochester, Minnesota

## Abstract

This cross-sectional study of US medical students assesses the association of COVID-19 intensity in the clinical learning environment with student burnout and perceptions regarding residency preparedness.

## Introduction

The prevalence of burnout among physicians increased in parallel with the COVID-19 pandemic.^1^ Despite disruptions in medical education, studies suggest that burnout among medical students has remained stable in recent years.^2,3^ We analyzed data from the 2019 to 2021 Association of American Medical Colleges (AAMC) Graduation Questionnaire, which is administered to graduating US medical school students, and data on COVID-19 cases and COVID-19–related deaths in the schools’ surrounding communities to explore the association between COVID-19 intensity in the clinical learning environment and student burnout and residency preparedness.

## Methods

In this cross-sectional study, we obtained population size and Centers for Disease Control and Prevention–recorded daily COVID-19 cases and COVID-19–related deaths for each medical school location in the US except Puerto Rico (eMethods in Supplement 1). Monthly means for cases and deaths were determined, converted to cases per 100 000 population, and divided into quartiles for 2020 and 2021 (eTable in Supplement 1). Schools were assigned quartiles for cases and deaths in 2020 and 2021. This information was shared with the AAMC, which matched the school quartile to the school of the responder, added demographic data previously provided by respondents, and deidentified the data. Because the data were deidentified, the Mayo Clinic and University of Colorado School of Medicine Institutional Review Boards deemed the study exempt from review and informed consent. We followed the STROBE reporting guideline.

The data set included responder demographics and responses to previously validated emotional exhaustion and disengagement Oldenburg Burnout Inventory (OBI) subscales^4^ and residency preparedness scale items (Cronbach α = 0.91). This scale included items about students’ perceptions of their skills, knowledge, values, and abilities.

Univariate differences were examined using 2-sided Kruskal-Wallis tests with statistical significance set at *P* < .05. Generalized linear regression models were conducted for 2020 and 2021 for the outcomes of interest and were adjusted for quartile of cases or deaths and age, sex, and race and ethnicity. All comparisons were performed using SAS, version 9.4 (SAS Institute Inc).

## Results

Response rates were 82.4% (16 427 of 19 935 students), 79.7% (16 253 of 20 390 students), and 79.9% (16 217 of 20 296 students) in 2019, 2020, and 2021, respectively. The mean (SD) exhaustion scores for these years were 12.7 (4.2), 12.7 (4.2), and 12.6 (4.2); disengagement scores were 5.4 (2.6), 5.4 (2.6), and 5.3 (2.6); and preparedness for residency scores were 40.3 (4.4), 40.4 (4.4), and 40.2 (4.7), respectively.

Associations were found between quartile of COVID-19 cases and the outcomes of interest. Higher 2020 quartile levels of COVID-19–related deaths in the primary learning environment were associated with small increases in mean disengagement scores and decreases in mean residency preparedness scores (Table 1). These associations persisted in multivariable linear regression analyses (Table 2).

**Table 1.** Emotional Exhaustion, Disengagement, and Residency Preparedness Scores of Graduating Medical Students by Quartile of COVID-19 Cases and COVID-19–Related Deaths in the Communities Surrounding Their Medical Schools, 2020 and 2021

	2020					2021				
	Quartile 1	Quartile 2	Quartile 3	Quartile 4	*P* value	Quartile 1	Quartile 2	Quartile 3	Quartile 4	*P* value
**COVID-19 cases^a^**										
Emotional exhaustion (range, 0-24)										
Median (IQR)	13.0 (10.0-15.0)	12.0 (10.0-15.0)	13.0 (10.0-15.0)	13.0 (10.0-15.0)	.01	12.0 (10.0-15.0)	12.0 (10.0-15.0)	12.0 (10.0-15.0)	12.0 (10.0-15.0)	.73
Mean (SD)	12.8 (4.16)	12.5 (4.21)	12.8 (4.19)	12.8 (4.23)		12.6 (4.20)	12.6 (4.14)	12.6 (4.15)	12.5 (4.23)	
Disengagement (range, 0-15)										
Median (IQR)	5.0 (4.0-7.0)	5.0 (4.0-7.0)	5.0 (4.0-7.0)	5.0 (4.0-7.0)	<.001	5.0 (4.0-7.0)	5.0 (4.0-7.0)	5.0 (4.0-7.0)	5.0 (4.0-7.0)	.07
Mean (SD)	5.3 (2.54)	5.2 (2.52)	5.5 (2.54)	5.4 (2.59)		5.4 (2.59)	5.3 (2.51)	5.3 (2.52)	5.3 (2.56)	
Residency preparedness (range, 9-45)										
Median (IQR)	41.0 (37.0-44.0)	41.0 (37.0-44.0)	41.0 (37.0-44.0)	41.0 (37.0-44.0)	.15	41.0 (36.0-45.0)	41.0 (36.0-45.0)	41.0 (36.0-45.0)	41.0 (37.0-45.0)	.01
Mean (SD)	40.3 (4.45)	40.5 (4.39)	40.4 (4.43)	40.3 (4.50)		40.2 (4.57)	40.2 (4.68)	40.1 (4.75)	40.4 (4.71)	
**COVID-19–related deaths^a^**										
Emotional exhaustion (range, 0-24)										
Median (IQR)	12.0 (10.0-15.0)	12.0 (10.0-15.0)	13.0 (10.0-16.0)	13.0 (10.0-16.0)	.01	12.0 (10.0-15.0)	12.0 (10.0-15.0)	12.0 (10.0-15.0)	12.0 (10.0-15.0)	.21
Mean (SD)	12.7 (4.24)	12.5 (4.12)	12.8 (4.22)	12.8 (4.21)		12.6 (4.23)	12.6 (4.24)	12.5 (4.09)	12.7 (4.16)	
Disengagement (range, 0-15)										
Median (IQR)	5.0 (4.0-7.0)	5.0 (4.0-7.0)	5.0 (4.0-7.0)	5.0 (4.0-7.0)	<.001	5.0 (4.0-7.0)	5.0 (4.0-7.0)	5.0 (4.0-7.0)	5.0 (4.0-7.0)	.49
Mean (SD)	5.2 (2.54)	5.3 (2.47)	5.4 (2.58)	5.5 (2.58)		5.3 (2.56)	5.3 (2.56)	5.3 (2.52)	5.4 (2.54)	
Residency preparedness (range, 9-45)										
Median (IQR)	42.0 (37.0-44.0)	41.0 (37.0-44.0)	41.0 (37.0-44.0)	41.0 (37.0-44.0)	<.001	41.0 (36.0-45.0)	41.0 (37.0-45.0)	41.0 (36.0-45.0)	41.0 (36.0-45.0)	.13
Mean (SD)	40.5 (4.47)	40.4 (4.34)	40.4 (4.50)	40.2 (4.44)		40.2 (4.60)	40.3 (4.71)	40.2 (4.65)	40.1 (4.76)	

^a^ Per 100 000 population.

**Table 2.** Multivariable Linear Regression of Associations Between Exhaustion, Disengagement, and Residency Preparedness Scores of Graduating Medical Students by Quartile of COVID-19 Cases and COVID-19–Related Deaths in the Communities Surrounding Their Medical Schools, 2020 and 2021^a^

	2020		2021	
	Estimate (SE)	*P* value	Estimate (SE)	*P* value
**COVID-19 cases**				
Disengagement score for each 1-point increase (2020, n = 14 985; 2021, n = 15 061)				
Quartile 1	1 [Reference]	NA	1 [Reference]	NA
Quartile 2	−0.05 (0.06)	.35	−0.10 (0.06)	.09
Quartile 3	0.22 (0.06)	<.001	−0.09 (0.06)	.14
Quartile 4	0.05 (0.06)	.44	−0.14 (0.06)	.02
Exhaustion score for each 1-point increase (2020, n = 14 955; 2021, n = 14 998)				
Quartile 1	1 [Reference]	NA	1 [Reference]	NA
Quartile 2	−0.32 (0.10)	.001	0.02 (0.10)	.82
Quartile 3	0.01 (0.10)	.89	−0.01 (0.10)	.89
Quartile 4	−0.05 (0.10)	.62	−0.10 (0.10)	.28
Residency preparedness score for each 1-point increase (2020, n = 15 401; 2021, n = 15 604))				
Quartile 1	1 [Reference]	NA	1 [Reference]	NA
Quartile 2	0.10 (0.10)	.31	−0.04 (0.11)	.73
Quartile 3	0.01 (0.10)	.90	−0.13 (0.11)	.24
Quartile 4	−0.05 (0.10)	.63	0.21 (0.10)	.04
**COVID-19–related deaths**				
Disengagement score for each 1-point increase (2020, n = 14 985; 2021, n = 15 061)				
Quartile 1	1 [Reference]	NA	1 [Reference]	NA
Quartile 2	0.10 (0.06)	.11	−0.05 (0.06)	.41
Quartile 3	0.12 (0.06)	.05	−0.03 (0.06)	.63
Quartile 4	0.23 (0.06)	<.001	0.04 (0.06)	.44
Exhaustion score for each 1-point increase (2020, n = 14 955; 2021, n = 14 998)				
Quartile 1	1 [Reference]	NA	1 [Reference]	NA
Quartile 2	−0.14 (0.10)	.15	0.04 (0.10)	.68
Quartile 3	0.12 (0.10)	.23	−0.13 (0.10)	.19
Quartile 4	0.15 (0.09)	.11	0.09 (0.09)	.36
Residency preparedness score for each 1-point increase (2020, n = 15 401; 2021, n = 15 604)				
Quartile 1	1 [Reference]	NA	1 [Reference]	NA
Quartile 2	−0.19 (0.10)	.07	0.14 (0.11)	.19
Quartile 3	−0.17 (0.10)	.09	0.06 (0.11)	.60
Quartile 4	−0.34 (0.10)	<.001	−0.12 (0.10)	.25

Abbreviation: NA, not applicable.

^a^ Per 100 000 population. Models were adjusted for sex, age at graduation, and race and ethnicity. For the entire cohort, the mean (SD) age at graduation was 27.7 (2.8) years; 48% were men and 52% were women. In terms of race and ethnicity, 0.7% of students were American Indian or Alaska Native; 25.5% were Asian; 7.1% were Black or African American; 8.5% were Hispanic, Latino, or of Spanish ancestry; 0.3% were Native Hawaiian or Other Pacific Islander; 63.1% were White; and 3.7%% were of other race or ethnicity (percentages exceed 100% because participants could select multiple categories). Race and ethnicity data were included in the data set because previous research has found associations between risk of burnout and medical student demographics.

## Discussion

This cross-sectional study found that burnout and perceptions of residency preparedness among US medical students did not worsen substantially from 2019 to 2021. Although an association was found between burden of 2020 COVID-19–related deaths and disengagement and perceptions of residency preparedness scores, the differences in scores were less than one half of 1 SD, a commonly used cutoff to determine significant changes.^5^

Study limitations include a modified version of the OBI and unknown minimally important differences for the measures. Despite a Cronbach α coefficient suggesting acceptable internal consistency, the validity of the residency preparedness scale has not been established. The COVID-19 cases and death rates during the last half of the fourth year may not completely reflect how the pandemic factored into graduating medical students’ experiences given variation in hospital resources and in-person educational experiences, unknown timing and location of away rotations, and other pandemic-related factors (eg, fewer social events, lockdowns). Whether stability in OBI and perceptions of residency preparedness scores reflect that medical school innovations^6^ mitigated medical student stress while preserving preparation for residency or if other factors explain these findings warrants further study.

## Notes

Supplement 1.
eMethods
**eTable.** Cutoff Scores by Quartile

Supplement 2.
Data Sharing Statement
