# Supplementary material for: Association of COVID-19 Intensity With Burnout and Perceptions of Residency Preparedness Among Medical Students
Source: JAMA Netw Open. 2023 Dec 13;6(12):e2347957. doi: 10.1001/jamanetworkopen.2023.47957 (PMC10719746; doi:10.1001/jamanetworkopen.2023.47957)
Supplement: Supplement 1. — eMethods eTable. Cutoff Scores by Quartile [file jamanetwopen-e2347957-s001.pdf]

Supplemental Online Content

Dyrbye LN, Brushaber D, West CP. Association of COVID-19 intensity with burnout and perceptions of residency preparedness among medical students. *JAMA Netw Open*. 2023;6(12):e2347957. doi:10.1001/jamanetworkopen.2023.47957

eMethods

eTable. Cutoff Scores by Quartile

This supplemental material has been provided by the authors to give readers additional information about their work.

© 2023 Dyrbye LN et al. *JAMA Network Open.*

eMethods

Addresses were obtained for US medical schools with 2019, 2020, and 2021 GQ data with exception of schools in Puerto Rico due to unavailable COVID case data. For each ZIP code a FIPS code was obtained (using publicly available data at https://www.kaggle.com/danofer/zipcodes-county-fips-crosswalk). FIPS codes were used to determine population size (available data at https://static.usafacts.org/public/data/covid-19/covid_county_population_usafacts.csv) and link to number of CDC recorded COVID cases (available at https://static.usafacts.org/public/data/covid-19/covid_confirmed_usafacts.csv) and COVID related deaths (available at https://static.usafacts.org/public/data/covid-19/covid_deaths_usafacts.csv) between February 1 and June 30, 2020 and January 31 and June 30, 2021 in the community surrounding the medical school (i.e., same zip code). These time periods were chosen as they correspond to when the AAMC GQ was available for medical students to complete. Monthly averages of COVID cases and deaths were determined, converted to cases per 100,000 population, and divided into quartiles for 2020 and 2021 shown in the below Table.

eTable. Cutoff Scores by Quartile

	Cases 2020	Deaths 2020	Cases 2021	Deaths 2021
Q1	104.04	2.26	322.17	5.5
Q2	162.44	4.84	403.91	7.74
Q3	272.72	15.61	576.78	10.81
Q4	720.16	67.11	952.69	23.9

Per 100,000 population

© 2023 Dyrbye LN et al. *JAMA Network Open.*
