# Supplementary material for: Association of COVID-19 Intensity With Burnout and Perceptions of Residency Preparedness Among Medical Students
Source: JAMA Netw Open. 2023 Dec 13;6(12):e2347957. doi: 10.1001/jamanetworkopen.2023.47957 (PMC10719746; doi:10.1001/jamanetworkopen.2023.47957)
Supplement: Supplement 2. — Data Sharing Statement [file jamanetwopen-e2347957-s002.pdf]

Data Sharing Statement

Dyrbye. Association of COVID-19 Intensity With Burnout and Perceptions of Residency Preparedness Among Medical Students. *JAMA Netw Open*. Published December 13, 2023. doi:10.1001/jamanetworkopen.2023.47957

Data
Data available: No

Additional Information
Explanation for why data not available: Data is owned by AAMC
